# Electrospun tube reduces adhesion in rabbit Achilles tendon 12 weeks post-surgery without PAR-2 overexpression

**DOI:** 10.1038/s41598-021-02780-4

**Published:** 2021-12-02

**Authors:** Gabriella Meier Bürgisser, Olivera Evrova, Dorothea M. Heuberger, Petra Wolint, Julia Rieber, Iris Miescher, Reto A. Schüpbach, Pietro Giovanoli, Maurizio Calcagni, Johanna Buschmann

**Affiliations:** 1 Division of Plastic Surgery and Hand Surgery, University Hospital Zurich, ZKF, Sternwartstrasse 14, 8091 Zurich, Switzerland; 2 Laboratory of Applied Mechanobiology, ETH Zürich, Vladimir-Prelog-Weg 1-5/10, 8093 Zurich, Switzerland; 3 Institute of Intensive Care Medicine, University Hospital Zurich, Sternwartstrasse 14, 8091 Zurich, Switzerland

**Keywords:** Cell biology, Molecular biology, Medical research

## Abstract

One great challenge in surgical tendon repair is the minimization of peritendinous adhesions. An electrospun tube can serve as a physical barrier around a conventionally sutured tendon. Six New Zealand White rabbits had one Achilles tendon fully transsected and sutured by a 4-strand suture. Another six rabbits had the same treatment, but with the additional electrospun DegraPol tube set around the sutured tendon. The adhesion formation to the surrounding tissue was investigated 12 weeks post-operation. Moreover, inflammation-related protease-activated receptor-2 (PAR-2) protein expression was assessed. Finally, rabbit Achilles tenocyte cultures were exposed to platelet-derived growth factor-BB (PDGF-BB), which mimicks the tendon healing environment, where PAR-2 gene expression was assessed as well as immunofluorescent staining intensity for F-actin and α-tubulin, respectively. At 12 weeks post-operation, the partially degraded DegraPol tube exhibited significantly lower adhesion formation (− 20%). PAR-2 protein expression was similar for time points 3 and 6 weeks, but increased at 12 weeks post-operation. In vitro cell culture experiments showed a significantly higher PAR-2 gene expression on day 3 after exposure to PDGF-BB, but not on day 7. The cytoskeleton of the tenocytes changed upon PDGF-BB stimulation, with signs of reorganization, and significantly decreased F-actin intensity. An electrospun DegraPol tube significantly reduces adhesion up to twelve weeks post-operation. At this time point, the tube is partially degraded, and a slight PAR-2 increase was detected in the DP treated tendons, which might however arise from particles of degrading DegraPol that were stained dark brown. PAR-2 gene expression in rabbit tenocytes reveals sensitivity at around day 10 after injury.

## Introduction

The surgical treatment of ruptured tendons still needs improvement strategies^1,2^. Surgeons are confronted with two major problems^3^. First, fibrosis may lead to a mechanically inferior tissue (scar), thus repaired tendons are prone for re-rupture^4^. Second, normal processes during healing, such as bleeding, infections, ischemia, foreign body reactions and fibrin deposition can result in adhesion formation to the surrounding tissue, which reduces the range of motion and hampers full functional performance^5^.

For minor adhesions, early active motion^6^, massage^7^, warmth or electrotherapy using acetic acid are successfully used^8^. However, there is often no successful therapy for patients with severe adhesions who do not only suffer from pain, but their limited range of motion impairs or even prohibits their daily activities.

Severe adhesions are reported most frequently for the *flexor digitorum profundus* (FDP) tendons as well as the *flexor digitorum superficialis* (FDS) of the hand in zone II, starting from the A1 pulley proximal edge to the distal insertion of FDS^9,10^. Nevertheless, also the Achilles tendon (AT) is often affected by adhesion formation^11^. A leaflet of fascia surrounds the AT, where adhesion may occur during the healing process.

Up to date, pharmacological strategies have been used, such as hyaluronic acid that acts like a lubricant and enables better gliding^12–15^ or 5-fluorouracil that blocks fibro-osseous fibroblast proliferation, associated with extrinsic healing^16^. Even anti-inflammatory drugs like ibuprofen showed to be a good anti-adhesion agent, inhibiting cyclooxygenases-1 and -2 and thereby keeping inflammation in check^17^.

Finally, the concept of a physical barrier between the repaired tendon and the surrounding tissue to prevent fibroblast penetration has been investigated. For that purpose, either natural materials like silk^18^ or collagen^19^ have been used or synthetic materials like water-borne polyurethane^20^. Such physical barriers may be realized as wraps around the lacerated tendons or as tubes, resembling tight-fitting mantles^21^. Important, these barrier materials should be biocompatible, degradable without cytotoxicity, lead to a full tissue integration and result in a low additional inflammatory response to the naturally occurring inflammation during the healing process. At best, such barriers are porous for unrestricted diffusion of nutrients and waste.

Previously, our research group has developed a synthetic electrospun nanofibrous tube of DegraPol (DP). There were no adverse cellular reactions, particularly no elevated inflammatory response, towards this implant placed around a fully-transsected rabbit AT^22^. In addition, we tuned the synthesis of DP to make it very elastic and thus more surgeon-friendly^21,23^. As a main result, we found a 20% reduction in the adhesion extent after 6 weeks, in two different models of hind leg immobilization; either adhesion provoking or adhesion reducing, realized by a change in ankle angle^24^.

In the study presented here, the main question was if the 20% reduction in adhesion is maintained over a longer period of time than the previously reported 6 weeks—because the tube is biodegradable, which might have an impact on long-term behaviour. Therefore, we analysed the adhesion formation 12 weeks post-operation by histology in the same way as previously reported^24^. The hypothesis was that although there was some degradation of the DP tube at 12 weeks (DP having a half-life time of half a year), there would still be a significant reduction in adhesion formation.

As a second aspect, we examined protease-activated receptor-2 (PAR-2) protein expression in tube-treated and contralateral non-treated rabbit AT. PAR-2 is an inflammation-related protein on the cell surface, potentially interesting in studies of fibrosis and scar formation^25^. PAR-2 staining was performed by immunohistochemistry as recently reported for rabbit lung and kidney tissue^26,27^. The hypothesis was that the application of a tube would not lead to an overexpression of PAR-2 because earlier studies had shown that macrophage cell density in tube-treated rabbit ATs had similar levels compared with non-treated corresponding tendons^24^.

Taken together, the two hypotheses of this study were:

(i) The partially degrading DP tube reduces adhesion formation significantly at 12 weeks post-operation.
(ii) The application of a DP tube does not lead to a PAR-2 overexpression during tendon healing up to 12 weeks.

## Materials and methods

### Synthesis of block block copolymer DegraPol

A biodegradable polyester urethane polymer (trade name DegraPol) with poly-hydroxy-butyrate as a crystalline segment and ε-caprolactone as a soft segment was synthesized according to an adapted protocol^21^. All chemicals were purchased from Sigma Aldrich, Switzerland, if not otherwise stated^21^. Briefly, for the synthesis of the block copolymer, 25 wt% of poly(3-(R)-hydroxybutyrate)-co-(ε-caprolactone)-diol (M_n_ = 2824 g mol^-1^), i.e. 2.5 g; and 75 wt% with M_n_ = 1000 g mol^-1^ poly(ε-caprolactone)-diol-co-glycolide (15 mol% glycolide 85 mol% ε-caprolactone), i.e. 7.5 g, were dissolved in 1.4-dioxane and dried by heating and refluxing the solvent over molecular sieves (pore size 0.4 nm) at 102 °C for 1.0 hour^21^. The reaction mixture was cooled to 83 °C. Then, the stochiometric amount of 2,2,4-trimethylhexane-diisocyanate (TMDI) was added. The stoichiometric amount was with respect to the two diols^21^. After 1 day of reaction, three portions dibutyltin dilaurate (20 ppm) were added within 1 day in order to reach molecular weight of 110 kDa^21^. The polymer was precipitated in dry ice cooled hexane isomers and subsequently purified via dissolution in chloroform and filtration over a silicagel 60 (Fluka, Switzerland) column. A second precipitation in cooled ethanol ended the process. The detailed procedure has been reported before^21^.

### Scaffold fabrication and characteristics

The electrospinning setup was assembled in-house and consisted of a syringe pump (Racel Scientific Instruments Inc., USA), a spinning head consisting of a central stainless steel tube (1 mm inner diameter and 0.3 mm wall thickness, Angst & Pfister AG, Switzerland), a cylindrical rotating aluminum mandrel for fiber collection (length: 100 mm, diameter: 4 mm) and a DC high voltage supply (Glassman High Voltage Inc., USA). A 12 wt% solution of the DegraPol (DP) in chloroform (Fluka, puriss., Switzerland) was prepared by dissolving the polymer under stirring overnight. Electrospinning voltage (15 kV) was applied with a high voltage supply between a needle (1 mm diameter) and the rotating cylindrical collector (20 cm apart from each other). The collector rotating speed was 500 rpm. The flow rate was 1 mL/ h. As-spun tubes (with randomly oriented fibres) were removed from the target by slightly swelling them with ethanol (Fluka, puriss., Switzerland) and then dried under vacuum at room temperature (RT). The overall mesh porosity P was calculated according to P = (1 − ρ/ρ_0_) × 100 [%], where ρ denotes the density of the electrospun mesh and ρ_0_ is the bulk density of the electrospun mesh determined gravimetrically using weights of precisely cut samples of defined area and thickness; P was 75.2 ± 0.4%. The wall thick-ness was 357.0 ± 17.7 µm as determined by means of scanning electron microscopy (SEM, FEI, Nova NanoSEM 450) as reported previously^23^. Mechanical properties for such tubes were investigated in-depth in a previous study^28^. Furthermore, biocompatibility had been confirmed previously as well^22^. The tubes had an inner diameter of 4 mm and were cut into pieces of 1 cm in length. Their degradation half-lives in water were determined to be approximately 0.5 years (information from the company *ab medica*, Italy).

### Animals

For the in vivo study, twelve female New Zealand White rabbits aged 12 to 16 weeks were used (Charles River, Research Models and Services, Germany). Only female rabbits were used because they do quarrel less than males, when hold in interconnected cages where they can meet each other. Animals were treated as reported before^29^. They were specific pathogen free (SPF). All animals were housed in pairs in two interconnected cages, each of them with a bottom area of 70 cm × 70 cm and a height of 62 cm (Indulab, Switzerland)^29^. The animals were maintained under controlled conditions: temperature 22 ± 1 °C, 45% relative humidity, 15 air changes per hour and a light/dark rhythm of 12 hours^29^. The rabbits had free access to water (automatic water supply), autoclaved hay and straw ad libitum and to standard pellet diet (Kliba Nafag, Nr. 3410, Provimi Kliba AG, Switzerland)^29^. Ethical approval was obtained for the experiments from the veterinary office of Zurich, Switzerland (reference numbers 193/2012 and 225/15). The veterinary office of Zurich has an animal welfare committee who approved our experiments. All experiments were carried out according to the relevant guidelines and regulations given by the veterinary office of Zurich and in compliance with the ARRIVE guidelines. Prior to surgery, all animals were acclimatized to their environment for two weeks.

### Achilles tendon repair

Before implantation, the DP tubes were sterilized with ethylene oxide at 37 °C. The rabbits received premedication with 65 mg/kg body weight ketamine and 4 mg/kg xylazine. A venous catheter was inserted in the marginal ear vein. The rabbits were intubated with Propofol i.v. 0.6–1.3 mg/kg. Anaesthesia was maintained with 1–2% isofluorane. In order to ensure systemic analgesia during the time of operation, 0.2–0.3 mg/kg body weight butorphanol (Dr. E. Graeub AG, Berne, Switzerland) was applied pre-operatively. The hind legs were cleaned with iodine. The AT exposure was obtained through a paratendineal incision of cutis, *subcutis* and *fascia*, according to a previous report^22^. The medial and lateral *M. gastrocnemius* of the AT complex were then sliced perpendicularly to the length of the tendon 2.0 cm above the *calcaneus* and the two tendon stumps were sutured (4-strand Becker suture) using a USP 4-0 polypropylene thread. In case of DP tube application, one of the two tendon stumps was pulled through the DP tube before suturing. Subsequently the wound was closed with a running suture (using a USP 6.0 polypropylene thread) of the fascia and interrupted skin. Immediately post-surgery, a Durogesic Matrix patch (Janssen-Cilag AG, Switzerland) was applied with 4.2 mg fentanyl per patch in order to provide analgesia for about 72 h with 25 µg/h Fentanyl. Postoperative treatment included a cast having an angle of 180° at the ankle for the first three weeks. The cast was well padded and it was changed after 3 weeks. The new cast had a smaller angle of 150°; as an adhesion inhibiting immobilization technique^24^. Nevertheless, this immobilization technique still allows adhesion formation, just on a lower level compared to the adhesion provoking model, where in contrast the ankle angle is not changed after 3 weeks^29^. Great attention was paid to make the casts not too tight so that they were tolerated well by the rabbits (they did not bite the cast). After another 3-week period, the second cast was removed. Twelve weeks post-surgery, the rabbits were euthanized in deep anaesthesia (100 mg/kg body weight ketamine and 4 mg/kg xylazine) with 80 mg/kg pentobarbital (Esconarkon *ad us. vet*., Switzerland).

### Treatment groups

The twelve rabbits were randomly divided into two groups. N = 6 was chosen based on previous findings^24^ with a power of 0.8 and α = 0.05. Six rabbits received a 4-strand Becker suture, the other six rabbits received the same suture, but in addition a DP tube tightly fitting around the suture. The counter legs for all twelve rabbits were not transsected and not treated (NT) and served as controls.

### Quantification of adhesion extent and histological stainings

After extraction, the AT specimens were immediately frozen at − 20 °C. After being thawed to room temperature (RT), they were paraformaldehyde-fixed (dehydrated, paraffin-embedded and sectioned into 5 µm thick slices, which were cross-sections in the DP tube region (perpendicular to the AT) according to previous protocols^22^. After de-paraffinizing with xylene and rehydrating the sections (descending gradient of ethanol), they were differently stained: Picosirius Red and Hematoxylin&Eosin (H&E) according to commonly established procedures, and PAR-2 immunohistochemical staining (details see below).

Picosirius Red stained sections were used to quantify the adhesion extent at 8 × magnification (Leica EZ4D microscope, Switzerland). Here, adhesion formation was quantified in five subsequent cross-sections separated by 2.0 mm according to a method by Tan et al*.*^30^. Analysis was done in a blinded fashion. The percentage of adhesion was calculated by the length of the contact region of the tendon under view with the surrounding tissue, divided by the total perimeter as reported previously^24^. The length of the contact region and the whole perimeter were determined using *synedra View 3* software (version 3.1.0.3).

Moreover, immunohistochemistry for PAR-2 was performed. Paraffin sections underwent an antigen retrieval (AR) step in 10 mM citrate buffer (pH 6.0) with 0.05% Tween-20 for 20 min at 95 °C. Next, sections were blocked in 5% donkey serum and 1% BSA in 1 × TBS for 1 h at RT. Afterwards, sections were incubated with mouse anti-PAR-2 antibody (Santa Cruz Biotechnology, sc-13504 (SAM11), 1:250 dilution) on Refine-kit (anti-Rabbit-Polymer) and histofine-Mouse Polymer (1:50 dilution) diluted in 3% bovine serum albumin (BSA) in 1xTBS overnight at 4 °C. Laboratory validation for PAR-2 antibody was reported earlier^26^.

Subsequently, for chromogenic immunohistochemistry of PAR-2 staining, samples were blocked with a 3% aqueous hydrogen peroxide solution, 10 min at RT and subsequently washed three times with 1xTBS. The protocol for PAR-2 staining has been reported previously^26^ and has been used here as well. The primary antibody detection was performed using a biotinylated anti-mouse IgG secondary antibody and streptavidin–horseradish peroxidase (HRP) (ZytoChem Plus HRP Kit Mouse; Zytomed Systems, Muttenz, Switzerland), followed by colorimetric detection using DAB substrate (DAB Substrate Kit High Contrast; Zytomed Systems, Germany) according to the manufacturer’s protocol. Afterwards, slides were washed in tap water and mounted using Faramount Aqueous Mounting Medium (Agilent). Fiji Image J 1.53a was used to determine brown intensity of PAR-2 protein expression, by the assessment of the red-to-green ratio colour intensity in the corresponding histograms, with n = 5 FOVs per section and n = 3 sections per group, leading to a total of 15 FOVs.

### Isolation of rabbit Achilles tenocytes and in vitro cell culture

Rabbit tenocytes were isolated from ATs of New Zealand White rabbits using the cell migration method (Approval by the veterinary office of Canton Zurich, reference number 255/15) as reported previously^28^. Briefly, tendons were extracted from the animals and washed with Hank’s Balanced Salt Solution (1 × HBSS with Ca^2+^ and Mg^2+^, Thermo Fisher Scientific, Rockford, IL, USA) with 200 µg/mL gentamicin (Biowest, Nuaillé, France) and 2.5 µg/mL amphotericin B (Biowest, Nuaillé, France). The tendons were cleaned from the surrounding tissue and the central part of the tendons was cut into very small pieces (< 2 mm) and washed three times in 1 × HBSS buffer. Afterwards, multiple tissue pieces were placed into each tissue culture plate (PrimariaTM, Corning, New York, NY, USA) and a drop of cell culture medium (Ham’s F12 (Biowest, Nuaillé, France), 10% FBS (Biowest, Nuaillé, France), 200 µg/mL gentamicin, and 2.5 µg/mL amphotericin B) was added onto each tissue piece. Tissues were allowed to attach onto the cell culture plates for 2 h at 37 °C and 5% CO_2_ before adding 10 mL of cell culture media in each plate^28^.

The plates with the tissues were not moved for the first 5 days, to decrease tissue detachment upon plate movement and to allow cells to start migrating out from the tissues. The first medium change was done after 5 days, and subsequently, the culture medium was changed every third day. After approximately 2 weeks, tissue pieces were removed from the plates, and cells were allowed to proliferate for 1 week more before cryopreservation. Cryopreserved rabbit tenocytes were thawed, resuspended in culture medium (Ham’s F12 with 10% FBS and 50 µg/mL gentamicin) and cultured at 37 °C and 5% CO_2_ with media being changed every second day. Tenocytes between passages 2 and 4 (P2–4) were used for all experiments. The same procedure has been reported before^31^.

Cells were stained for F-actin cytoskeleton (Phalloidin-Alexa488) and for α-tubulin (Anti-alpha tubulin antibody [TU-01] (ab7750)) when 80% confluency was reached after a cultivation period of 1 week in Ham’s F12 culture medium with 10% FBS and 50 µg/mL gentamicin. Specifically, cells were washed with PBS, fixed with paraformaldehyde (4%) for 15 min at RT and rinsed two times with PBS after aspiration of the fixative.

Cells were then permeabilized with 0.5% Triton-X 100 in PBS for 20 min and afterwards rinsed two times with PBS after aspiration of the solution. Subsequently, cells were incubated with the primary antibody for α-tubulin (diluted 1:200, final concentration 5 μg/mL) overnight at 4 °C. Then, cells were incubated with a master mix, composed of the second antibody (Alexa647-anti mouse (A21238), final concentration 10 μg/mL), phalloidin (20 μg/mL) and DAPI (4′6-diamidino-2-phenylindole di-lactate, Sigma-Aldrich, final concentration 5 μg/mL) in phosphate buffered saline (PBS), pH 7.4, for 1 h at RT, protected from light. Samples were rinsed three times for 5 min with PBS before microscopy (Leica Microscope 6000 B, Leica Camera AG, Germany; with laser405 for DAPI; laser488 for Phalloidin and laser633 for Alexa647). Images were analyzed for size of the cells (i.e. length, n = 35 cells), aspect ratio (n = 35), F-actin green intensity (n = 30 Fields of View, FOVs) and α-tubulin red intensity (n = 30 FOVs), respectively, using histograms of the corresponding FOVs in Fiji Image J 1.53a.

### Real-time PCR

In order to determine the effect of PDGF-BB supplementation on the gene expression of tenocytes in vitro over time, rabbit tenocytes were isolated and cultured as described under section *2.7.* and seeded into 12-well plates (TPP, Trasadingen, Switzerland, growth area per well: 3.60 cm^2^) with a density of 40,000 cells/ well, according to previous protocols^31^. Cells were allowed to attach overnight before the medium was exchanged to medium with PDGF-BB (20 ng/mL) or without any supplementation (Ham’s F12, 10% FBS, 50 µg/mL gentamicin). Desired PDGF-BB concentration was added freshly to the medium every time during medium exchange. The medium was changed every second day, and cells were stimulated with PDGF-BB for a total of 14 days. Samples were collected after 3, 7 and 14 days. Rabbit tenocytes from four different animals were used (n = 4) and experiments were performed in triplicates for each rabbit^31^.

Furthermore, tenocytes were exposed for 2 h in serum-free medium to different inhibitors in a concentration of 40 µM, before the medium was exchanged to medium with PDGF-BB (20 ng/mL) or without any further supplementation (Ham’s F12, 5% FBS, 50 µg/mL gentamicin). The inhibitors used were SB203580: inhibitor for MAPK (mitogen-activated protein kinase); LY-294002: inhibitor for PI3K (phosphatidylinositol 3-kinase) and PD153035; inhibitor for EGFR (epidermal growth factor receptor). After 3 days, PAR-2 gene expression was assessed.

At the respective time point, total RNA was isolated using the RNeasy Plus Mini Kit (Qiagen, Hilden, Germany, Switzerland ) with RNase-free DNase treatment (Qiagen, Hilden, Germany), following the manufacturer’s protocol. For reverse transcription (RT), 500 ng of total RNA was reverse transcribed into cDNA in a reaction volume of 20 µL using the iScript Advanced cDNA Synthesis Kit (Bio-Rad, Cressier, Switzerland), according to a previous report^31^. Real-Time PCR reactions were performed on the resulting cDNA samples (5 ng cDNA per reaction), using the CFX Connect Real-Time PCR Detection System (Bio-Rad, Cressier, Switzerland) and SsoAdvanced SYBR Green Supermix (Bio-Rad, Cressier, Switzerland). The PCR reactions were incubated at 95 °C for 3 min, followed by 39 cycles of 95 °C for 10 s and 62 °C for 30 s. All samples were run in technical duplicates. The primer sequences for PAR-2 forward were (5′–3′): TTC CCG GCC TTC CTC ACG G and reverse CTT CTG GGA GCT GAG GGA C. All primers were synthesized by Microsynth, Balgach, Switzerland. Relative expression analysis was performed using the comparative 2^−∆∆CT^ method with gapdH as a reference gene, which was stable over the two conditions analyzed. Results are presented as fold change normalized to control, i.e., compared to samples cultivated without PDGF-BB (set to 1).

### Statistical analysis

Data were analysed with StatView 5.0.1 and SPSS Statistics Version 25. Data were checked for normal distribution with Kolmogorov-Smirnow and Shapiro–Wilk tests. Moreover, variance homogeneity was checked with Levene’s test. Because data were normally distributed and had no significant different variances, parametric one-way analysis of variance (one-way ANOVA) was conducted for differences between more than two groups. Unpaired t test was performed for differences between two groups. Pairwise comparison probabilities (*p*) were calculated using the Bonferroni posthoc test. *p* values < 0.05 were considered significant and indicated by *; for *p* < 0.01 (**) and for *p* < 0.001 (***). Values were expressed as means ± standard deviations.

## Results

### Degradation of electrospun DegraPol fiber mesh

**Figure 1:**
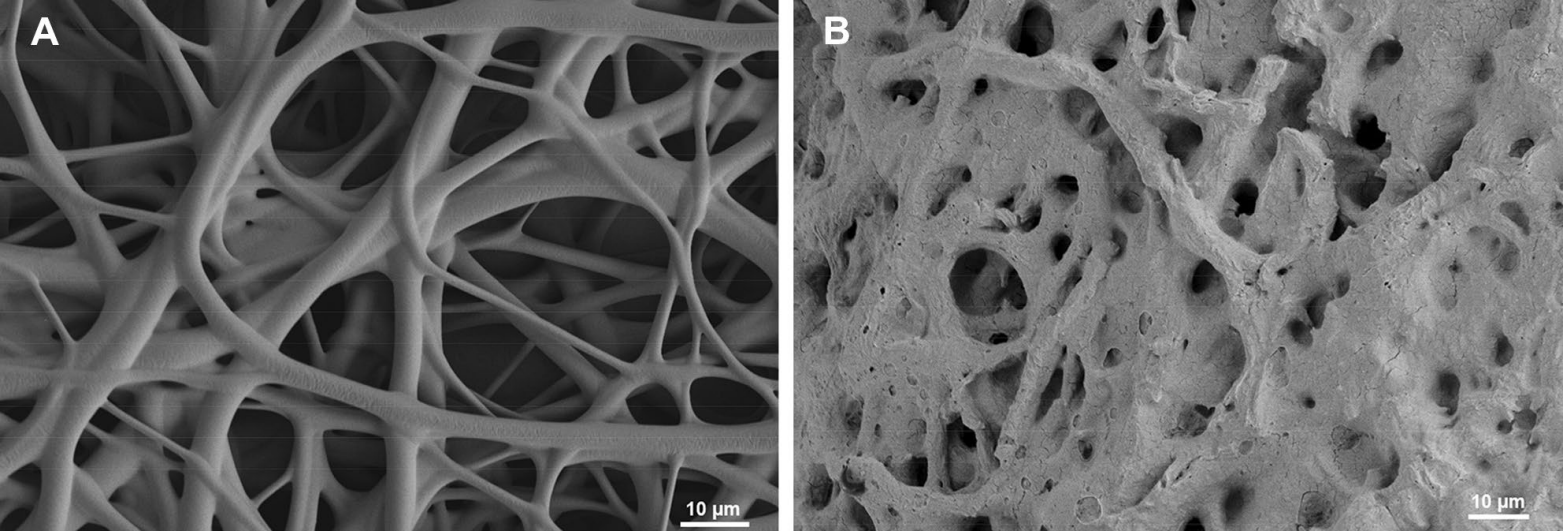
SEM images of electrospun DP fiber mesh freshly produced (**A**) and after 12 weeks in vivo as implant material intended to reduce adhesion formation (**B**).

DegraPol is a biodegradable polymer, which degrades slowly by hydrolysis over time. Its half-life is approximately half a year (information from *ab medica*, Italy, the company manufacturing DegraPol). Figure 1 shows two scanning electron microscopy (SEM) images. In Fig. 1A, a freshly produced DP fiber mesh is shown. Figure 1B shows the DP fiber mesh after 12 weeks in vivo in the rabbit full transection AT model. Compared to freshly produced DP fiber meshes, the fibers had merged to some extent. Some big pores were still visible well after 12 weeks in vivo, however, compared to a freshly prepared DP fiber mesh, many small pores had vanished at that time point due to degradation and fiber fusion.

### Adhesions at 12 weeks post-operation

The influence of a pure DegraPol tube was assessed by histological analysis of the close contact region between the tendon and the surroundings, and tube-treated tendons were compared with tendons repaired without tube (control: contralateral side, no treatment at all).

**Figure 2:**
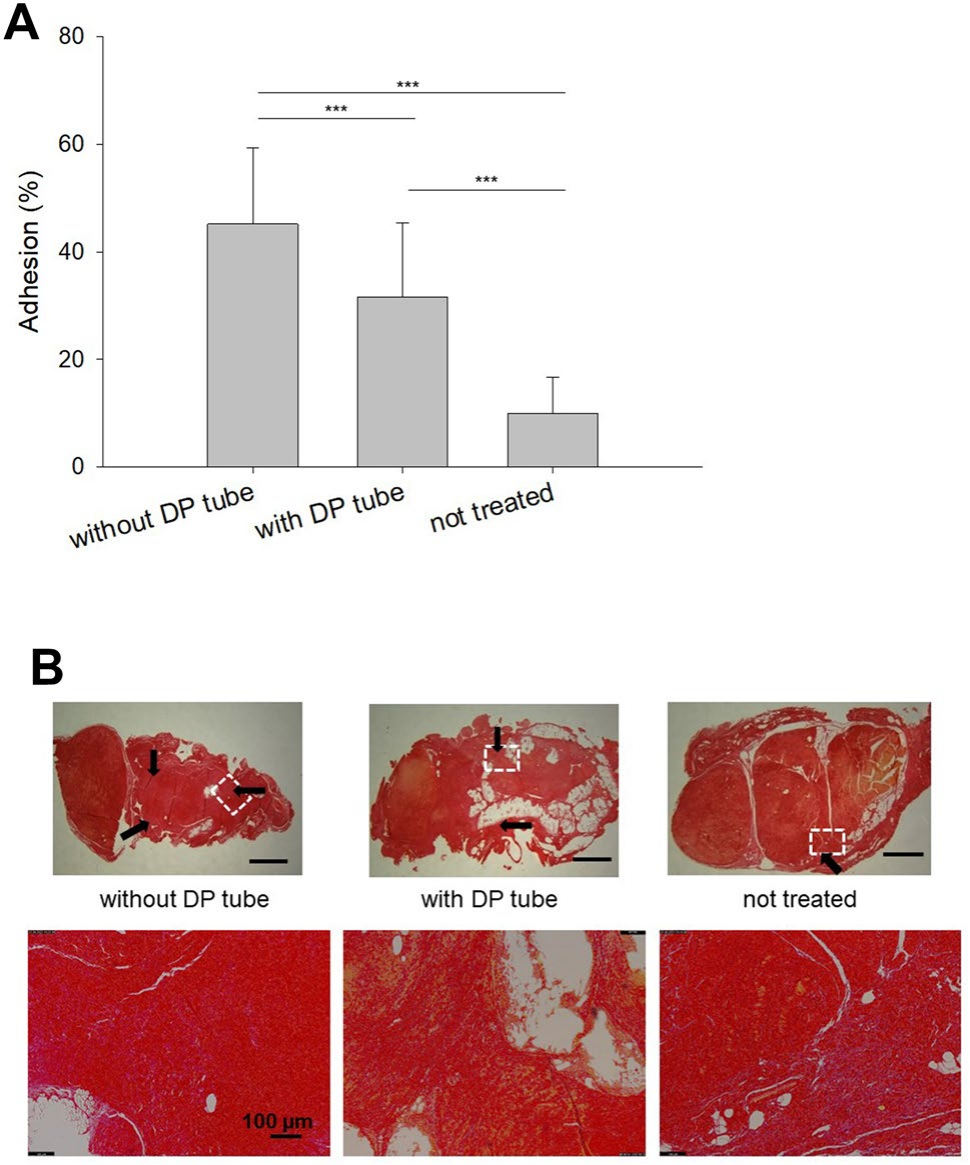
Adhesion formation as assessed by histology via contact region to the surrounding tissue. Adhesions were determined at 12 weeks. Quantitative determination: percentage of contact region (**A**); typical Picosirius Red stained cross-sections, including white dashed areas that are magnified below each image (**B**). *Key*: DP = DegraPol. Stars, with p-values, indicate pairwise comparison probability: *p* < 0.05 (*), *p* < 0.01 (**) and *p* < 0.001 (***). Scale bars indicate 500 µm. Arrows depict areas of adhesion (contact to surrounding tissue).

The application of such a tube pulled over a conventional 4-strand suture led to a significant reduction in adhesion formation at 12 weeks, when compared to the same suture without tube (Fig. 2). However, compared to time point 6 weeks, where the tendons had been immobilized up to this time point, adhesions were generally higher at 12 weeks post-operation for both groups, with and without tube, respectively (SI Fig. 1). The absolute adhesion extent, although significantly lower for the tube-treated group, was very similar to the results determined at 3 weeks post-operation (SI Fig. 1).

### PAR-2 protein expression in tendons treated with DP tube compared to contralateral side

**Figure 3:**
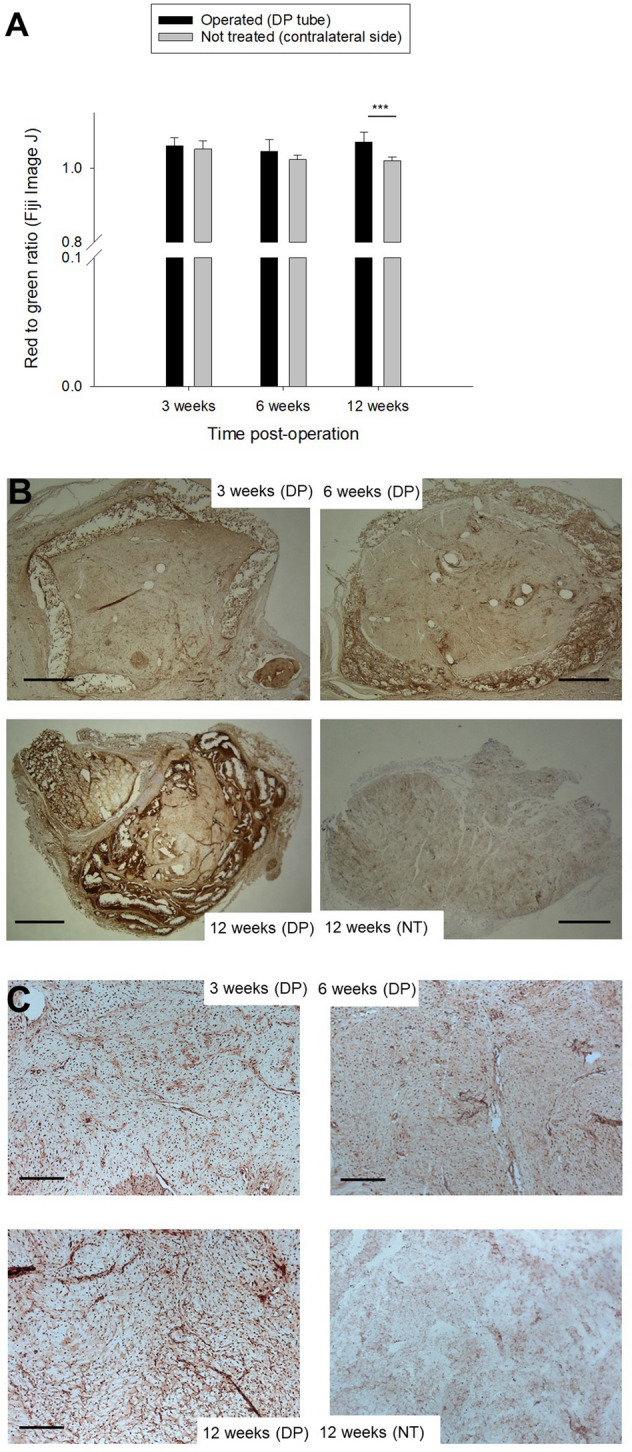
PAR-2 protein expression in DP treated tendon sections (n = 3) compared to contralateral side (no treatment; n = 3); quantified by the red/green ratio in histograms with 5 FOVs per section (**A**) and representative samples stained for PAR-2 (**B**), with areas of higher magnification, taken from the middle of the AT (**C**). The dark brown staining at 12 weeks does not come from an overexpression of PAR-2 by the tenocytes; it is the partially degraded DegraPol polymer that stains like this (Supporting Information SI Fig. 3). Nevertheless, the mid portion of the AT showed a significant slight increase in PAR-2 expression (+ 4.9%). Scale bars indicate 500 µm; in inserts they indicate 100 µm. Stars, with p-values, indicate pairwise comparison probability: *p* < 0.05 (*), *p* < 0.01 (**) and *p* < 0.001 (***).

Excessive pain signaling in lacerated human ATs had been partially explained by PAR-2 overexpression before^32^. DP treated tendon sections were therefore stained for PAR-2 protein expression by immunohistochemistry and compared to sections of the contralateral untreated tendons (NT). For the first two time points (experiments at 3 and 6 weeks performed previously^24^, only stainings performed here), PAR-2 expression between DP and NT was similar and there was no statistically significant difference. For the time point 12 weeks, however, DP treated specimen had a significantly higher PAR-2 expression compared to contralateral ATs, based on computationally assessed red-to-green ratios (Fig. 3 and SI Fig. 2). It has to be emphasized, however, that this significant increase was only an increase of 4.9%.

### PAR-2 gene expression in PDGF-BB stimulated rabbit tenocytes

**Figure 4:**
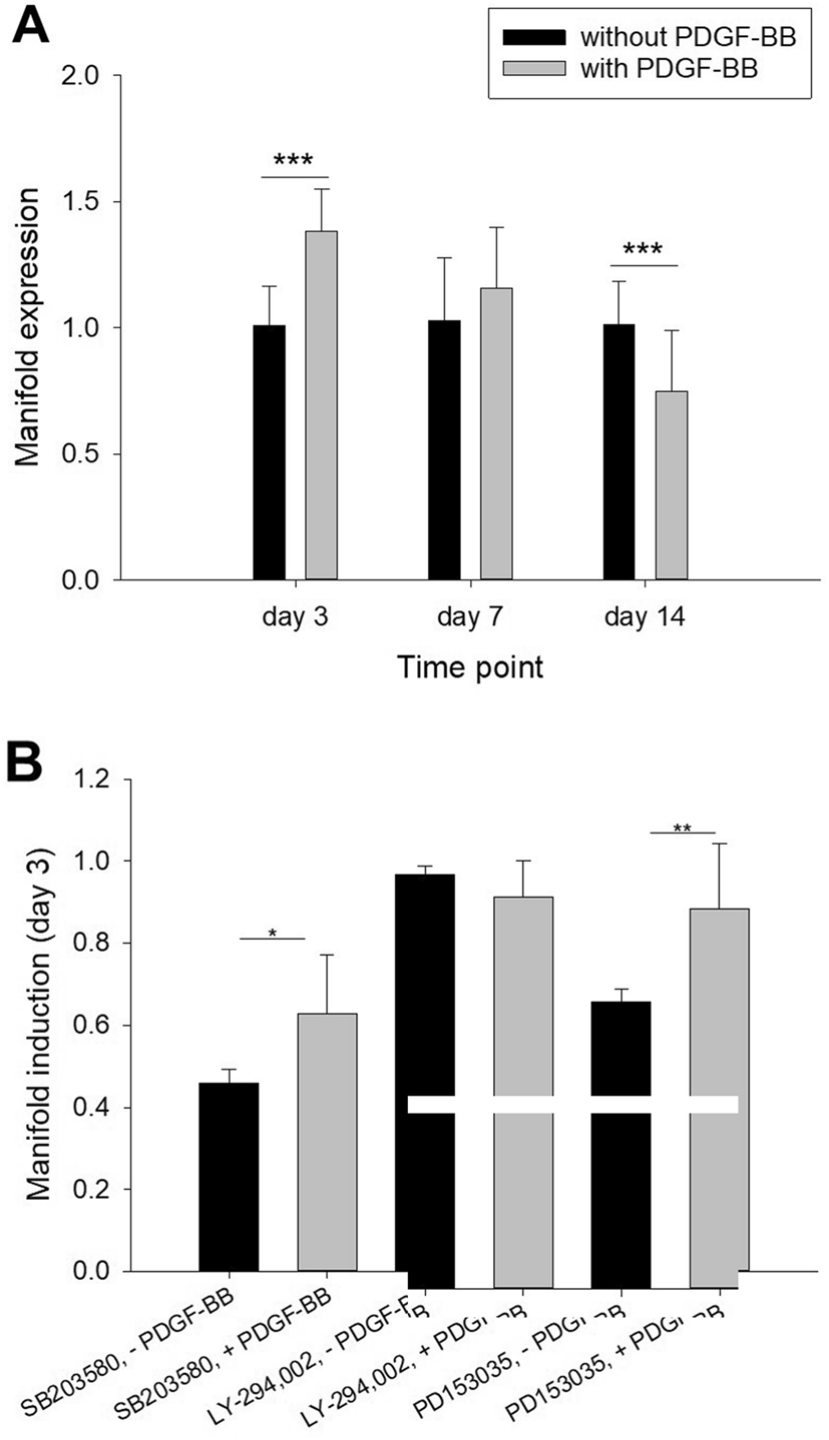
PAR-2 gene expression with 20 ng/mL PDGF-BB and without PDGF-BB after 3, 7 and 14 days under in vitro culture; manifold expression compared to cell culture without PDGF-BB; for tenocytes of four rabbits. (**A**). Manifold PAR-2 gene expression in a 3-day experiment, using different inhibitors in a 40 µM concentration and 5% FBS (**B**). Key: SB203580 = inhibitor for MAPK; LY-294002 = inhibitor for PI3K; PD153035 = inhibitor for EGFR (same family as PDGFR); DMSO = dimethyl sulfoxide (solvent of inhibitors); Medium = basal culture medium (with 10% FCS).

As PAR-2 protein expression was only significantly overexpressed at 12 weeks post-operation and with only a 5-percent increase (Fig. 3A), we furthermore checked under in vitro conditions if there was also an effect of PDGF-BB on the gene expression of PAR-2 at 3, 7 and 14 days, which are earlier time points than the shortest in vivo end-point of three weeks. While a 3-day exposure to PDGF-BB led to a significant upregulation of PAR-2 expression in rabbit tenocytes extracted from four donor ATs, after 7 days it had returned to baseline values and further downregulated at 14 days (Fig. 4A). Moreover, gene expression of PAR-2 was assessed after exhibition to different inhibitors and stimulation with PDGF-BB (Fig. 4B). While inhibition of MAPK as well as inhibition of EGFR did not affect PAR-2 gene upregulation, inhibition of PI3K resulted in approximately the same PAR-2 gene expression with and without PDGF-BB; indicating a transactivation of PAR-2 and PDGFR via PI3K.

### Cytoskeletal reorganization

**Figure 5:**
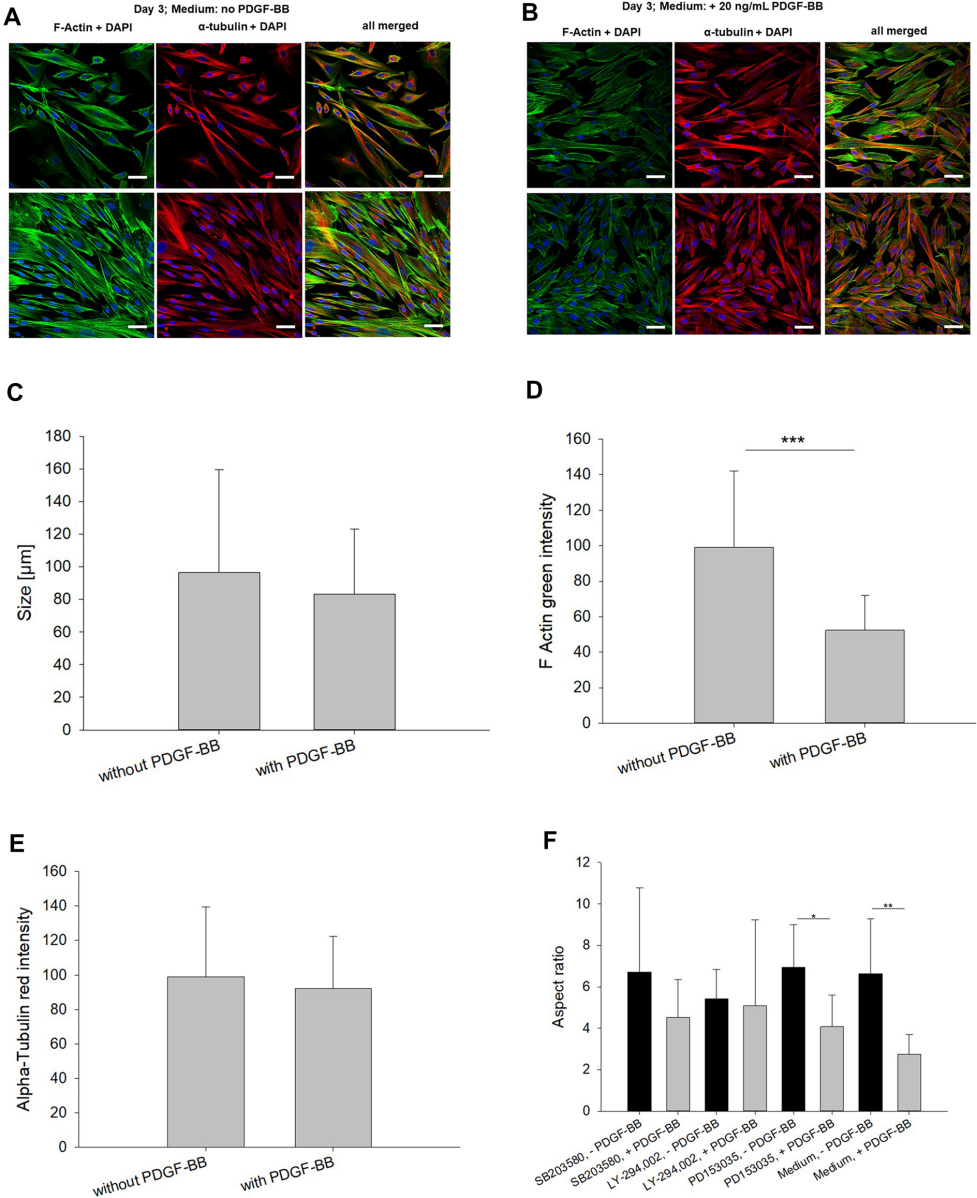
Images of tenocyte cultures without PDGF-BB (**A**) and with 20 ng/mL PDGF-BB (**B**) on day 3, showing F-actin (green), α-tubulin (red) and DAPI (blue, cell nuclei). PDGF-BB accompanying PAR-2 induction changes the F-actin cytoskeleton. Scale bars are equivalent to 50 µm. Achilles tenocytes of one rabbit are shown in the upper row, and of another rabbit in the lower row. Size of cells (**C**), F-actin green intensity (**D**) and α-tubulin red intensity (**E**), and aspect ratio of tenocytes on day 3 after addition of an inhibitor (40 µM, 5% FBS) with or without PDGF-BB (**F**). Key: SB203580 = inhibitor for MAPK; LY-294002 = inhibitor for PI3K; PD153035 = inhibitor for EGFR; DMSO = dimethyl sulfoxide (solvent of inhibitors); Medium = basal culture medium DMEM (with 10% FCS).

Going along with the PAR-2 upregulation in gene expression under PDGF-BB exposure, rabbit Achilles tenocytes underwent a cytoskeleton reorganization (Fig. 5). As can be seen, a 3-day exposure to 20 ng/mL PDGF-BB, changed the F-actin skeleton, with disruption and loosening of the F-actin filaments, which was accompanied by a significantly lower F-actin green intensity (Fig. 5D). In contrast, the α-tubulin filaments did not change very much, with only few of the filaments being loosened and approximately the same α-tubulin red intensity (Fig. 5E). However, a contraction of the tenocyte cytoskeleton was seen under PDGF-BB supplementation, where the aspect ratio changed significantly and the cells got (Fig. 5F). Moreover, it was observed, that the aspect ratio did not change when a PI3K inhibitor was applicated; in this case, aspect ratio was similar with and without PDGF-BB supplementation (Fig. 5F).

## Discussion

During tendon healing, the formation of fibrous adhesions to the surrounding tissue is a major problem, resulting in a restricted range of motion^33^. The etiology of adhesion formation roots in the destruction of the basement membrane, a cell-retentive layer on the tendon surface, where cells can exit upon damage and finally lead to an adhesion by deposits of extracellular matrix components^34^. Several anti-adhesion approaches have been developed recently, such as electrospun tubes with hyaluronic acid to address deep flexor tendons ruptures in the sheep model^35^; collagen-based (ElastiCo) barrier material to inhibit adhesion in the rat AT model^11^ or a cell-hydrogel fiber composite, mimicking the synovial sheath, promoting the endogenous hyaluronic acid formation, and enhancing the lubricating effect^36^.

In the past, we have developed a polymer tube that was placed around a conventionally sutured rabbit AT^22^. As there were no adverse cellular effects towards the polymer DegraPol (pH neutral and biodegradable)^24^, the synthesis of the polymer was adapted to yield a highly elastic tube^21^. This enabled the surgeon to put it over the wound site easily. The tube reduced adhesion formation by approximately 20% at 3 and 6 weeks post-implantation^24^.

Here, we performed rabbit AT full transections with conventional four-strand sutures with or without tubes for 12 weeks. The main finding is that even after partial and very well visible degradation of the polymer (Fig. 1 and SI Fig. 3), the polymer tube still acted anti-adhesive with a significant reduction of adhesion formation even after 12 weeks (Fig. 2), without any microvessel formation (SI Fig. 4). Compared to the time point 6 weeks, however, the absolute adhesion extent, as assessed by histological analysis, was higher at 12 weeks (SI Fig. 1A). After removal of the casts at 6 weeks, adhesions were obviously partially rebuilt, despite the fact that the rabbits were able to freely move in their cages until 12 weeks post-operation.

Furthermore, staining intensity did not vary much for PAR-2 protein expression at 3 and 6 weeks post-surgery; it was approximately the same for tendons with the tubes compared to non-treated tendons (Fig. 3). However, at 12 weeks post-operation, DP treated tendons had a significant, slightly higher PAR-2 staining intensity in the mid-portion of the tendon compared to the contralateral side, although the red-to-green ratio increased by only by 4.9%. This opens the discussion if PAR-2 was overexpressed due to some potential late inflammatory reaction here, which was not yet established at 3 and 6 weeks^37^.

However, as the partially degrading DP polymer exhibited a non-specific strong brown staining that was not related to any tenocytes (SI Fig. 3), another explanation for the + 4.9%-increase in PAR-2 protein expression (12 weeks) is that small particles from the degrading DP polymer had intruded the tendon proper during remodeling and had evoked a significant increase in the brown colour besides the tenocytes expressing PAR-2. Inflammatory response is usually an early event during tendon healing, occurring immediately after injury^38^, making the finding of a PAR-2 overexpression as late as 12 weeks particularly special and demanding for further explanations. Hence, it is highly probable that this small, though significant, increase in brown colour intensity is an artefact.

PAR-2 expression has been reported in tendinopathy before^32^. Besides PAR-2, there are also PAR-1, PAR-3 and PAR-4^39^. For human AT biopsies, it was found that PAR-1 and PAR-4 expressions were primarily found in the nerves of those biopsies, while PAR-2 expression was particularly associated with the tenocytes. Moreover, all four PARs (PAR-1 to PAR-4) were expressed in vessels that usually occur to a greater extent in damaged and healing tendons compared to healthy ones which are rather avascular. Here, we focused on PAR-2 expression in the mid portion of the healing AT, in other words where originally the transection had been set during the operation. As PAR-2 expression was only increased at 12 weeks and not at 3 and 6 weeks, we checked if PAR-2 was overexpressed at earlier time points during tendon healing (earlier than 3 weeks), when inflammation is usually more prominent^40,41^.

In that sense, a short-time simple in vitro model was created to mimic earlier time points than 3 weeks. Tenocytes harvested from four rabbit ATs were cultivated in vitro and exposed to 20 ng/mL of PDGF-BB, a growth factor that is released from platelets during tendon healing and which reaches its highest concentration around 7 days after the laceration^42^. At 3, 7 and 14 days after exposure to PDGF-BB, we assessed PAR-2 gene expression to get insight into the tenocytes’ response to such a typical growth factor of the tendon healing, mimicking the healing environment in vitro at least partially. It was found that at 3 days PAR-2 gene expression was significantly enhanced with PDGF-BB, while at 7 days, gene expression of PAR-2 was similar with and without PDGF-BB and even decreased after 14 days for the condition with PDGF-BB (Fig. 4).

Because the growth factor reaches its highest concentration around 1 week after the laceration in vivo, our 3-day experiment resembles a corresponding in vivo situation of around 10 days post-operation (7 days + 3 days); while the 7-day experiment resembles an in vivo situation of around 14 days post-operation (7 days + 7 days). The last in vitro time point (14 days) corresponds therefore to 21 days post-operation (7 days + 14 days). For the time point at 21 days, in vivo results showed that the PAR-2 protein expression was approximately the same with or without operation, so the in vitro findings (at 14 days) correspond well to the in vivo results (at 3 weeks); and do not deviate from our speculation that at 3 weeks post-operation an eventual PAR-2 overexpression is already “over”.

Interestingly, tenocytes reacted towards PDGF-BB exposition by changing their F-actin cytoskeleton. Although PDGF-BB has been reported to activate actin polymerization in pulmonary hypertension^43^, there was a clear reorganization of the F-actin filaments with some loosening between the tenocytes in our in vitro experiment (Fig. 5A and B). The F-actin green intensity was significantly reduced as well (Fig. 5D). As for the α-tubulin, changes were less visible and intensities were similar (Fig. 5E).

It is proposed that the observed reorganization of the F-Actin cytoskeleton under these in vitro conditions is caused by the phosphorylation of Akt, which is activated by PDGF-BB as shown previously^28^. We speculate that the signaling goes via a pathway of transactivation of PDGFR receptor^44^ as well as PAR-2, because higher PAR-2 expression had been reported by PDGF-BB for fibroblasts^45^. Furthermore, PAR-2 activation induced PDGFR receptor^46^. Some evidence for this proposed transactivation was found in this study here, when we inhibited PI3K. Under PDGF-BB stimulation and inhibition of PI3K, PAR-2 gene expression was not upregulated and showed a very similar PAR-2 gene expression level as the control without PDGF-BB in the presence of PI3K inhibitor (Fig. 4B); going along with similar aspect ratios of the cells (Fig. 5F). This indicates that the transactivation of PAR-2 and PDGFR might go via PI3K. In contrast, inhibition of MAPK still led to a PAR-2 gene upregulation (Fig. 4B).

Moreover, PAR-2 has been reported to be affecting cytoskeletal organization of epithelial cells^47^ and driving cytoskeletal dynamics in keratinocyte differentiation^48^. Additionally, PAR-2 is very similar to PAR-1, for which cytoskeletal reorganization effects have been reported as well^49^. As we observed that tenocytes were getting rounder under PDGF-BB supplementation, losing their slenderness with disruption of the typical tenocyte-tenocyte connections (Fig. 5A and B), going along with PAR-2 gene upregulation on day 3 (Fig. 4A), the regulation of gap junction protein Connexin 43 has to be mentioned as well. For PAR-1 which is similar to PAR-2, regulation of Connexin 43 has been reported^50^, yet another indication for a PAR-2 crosstalk with PDGFR. Further verifying mechanistic experiments will be, however, the topic of future investigations.

## Conclusion

In summary, we could demonstrate that an electrospun DegraPol tube acts as an efficient physical barrier at 12 weeks post-surgery despite partial degradation. This is a time point where usually patients can fully return to daily activities. Moreover, slight PAR-2 overexpression in the healing tendon was found only at 12 weeks post-operation (+ 4.9%),—a very late time point and highly improbable for inflammatory response to occur. Because degrading DP stained non-specifically dark brown during PAR-2 immunohistochemistry, one explanation for this slight increase of PAR-2 intensity at 12 weeks might be (dark) DP particles invading the AT during remodeling, thus being only an artefact.

In an in vitro tendon healing model, with tenocytes exposed to the growth factor PDGF-BB, a closer look at the PAR-2 dynamics revealed that PAR-2 gene expression was significantly higher in the presence of PDGF-BB after a 3-day exposure. Like this, it seems that PAR-2 overexpression by tenocytes was already over under in vivo conditions at the first time point analyzed here (3 weeks post-surgery). From these in vitro findings we conclude that PAR-2 overexpression must have occurred around at day 10 after the operation, supposed that maximum PDGF-BB concentration in the healing tendon occurred around 7 days post-surgery^42^.

## Supplementary Information

Supplementary Information.
