# Supplementary material for: Electrospun tube reduces adhesion in rabbit Achilles tendon 12 weeks post-surgery without PAR-2 overexpression
Source: Sci Rep. 2021 Dec 2;11:23293. doi: 10.1038/s41598-021-02780-4 (PMC8639666; doi:10.1038/s41598-021-02780-4)
Supplement: Supplementary file 1 — Supplementary Information. [file 41598_2021_2780_MOESM1_ESM.docx]

**Supporting Information**

**Electrospun tube reduces adhesion in rabbit Achilles tendon 12 weeks post-surgery without PAR-2 overexpression**

Gabriella Meier Bürgisser^1^, Olivera Evrova, Ph.D. ^1,2^, Dorothea M. Heuberger, Ph.D. ^3^, Petra Wolint^1^, Julia Rieber^1^, Iris Miescher^1^, Reto A. Schüpbach, M.D. ^3^, Pietro Giovanoli, M.D.^1^, Maurizio Calcagni, M.D.^1^ and Johanna Buschmann, Ph.D.^1^*

^1^Division of Plastic Surgery and Hand Surgery, University Hospital Zurich, Sternwartstrasse 14, 8091 Zurich, Switzerland

^2^Laboratory of Applied Mechanobiology, ETH Zürich, Vladimir-Prelog-Weg 1-5/ 10, 8093 Zurich, Switzerland

^3^Institute of Intensive Care Medicine, University Hospital Zurich, Sternwartstrasse 14, 8091 Zurich, Switzerland

* Corresponding author:

*Dr. Johanna Buschmann*

University Hospital Zurich, ZKF, Division of Plastic Surgery and Hand Surgery

Sternwartstrasse 14, 8091 Zurich, Switzerland

Phone: +41 44 255 98 95

Fax: +41 44 255 50 47

e-Mail: [johanna.buschmann@usz.ch](mailto:johanna.buschmann@usz.ch)


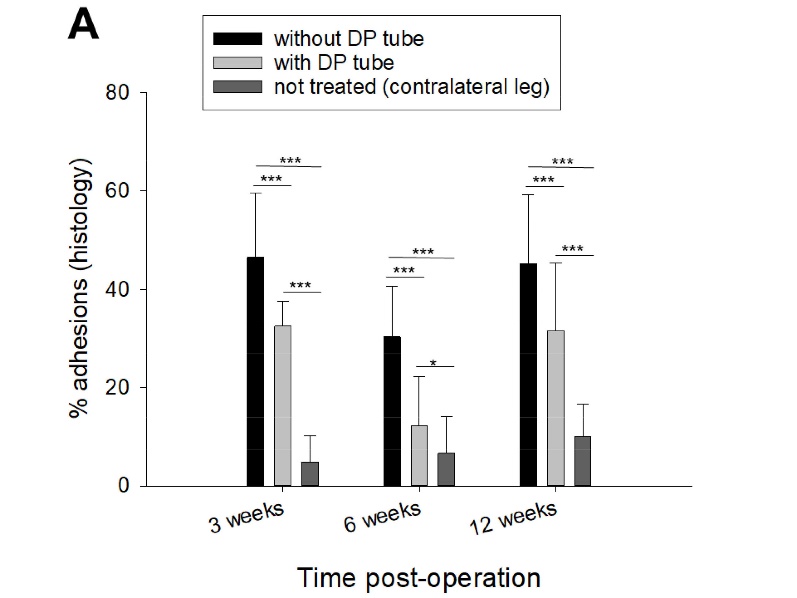


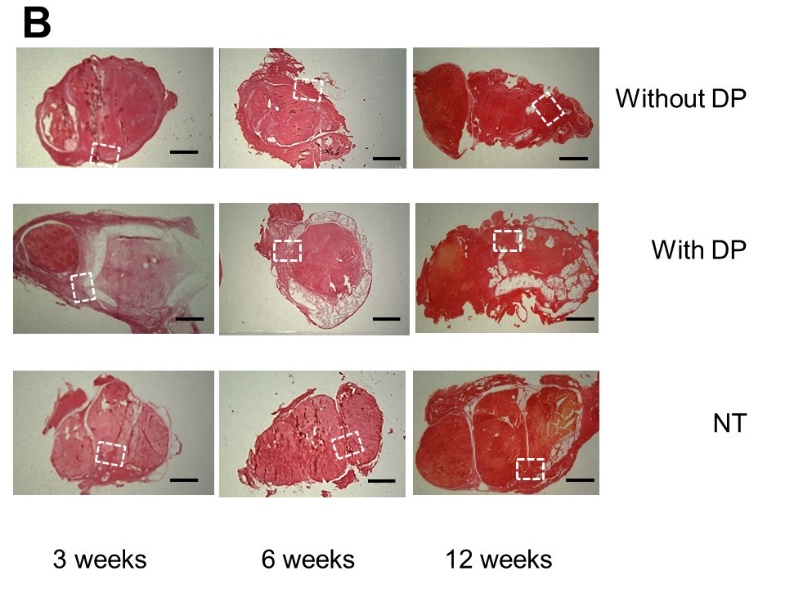


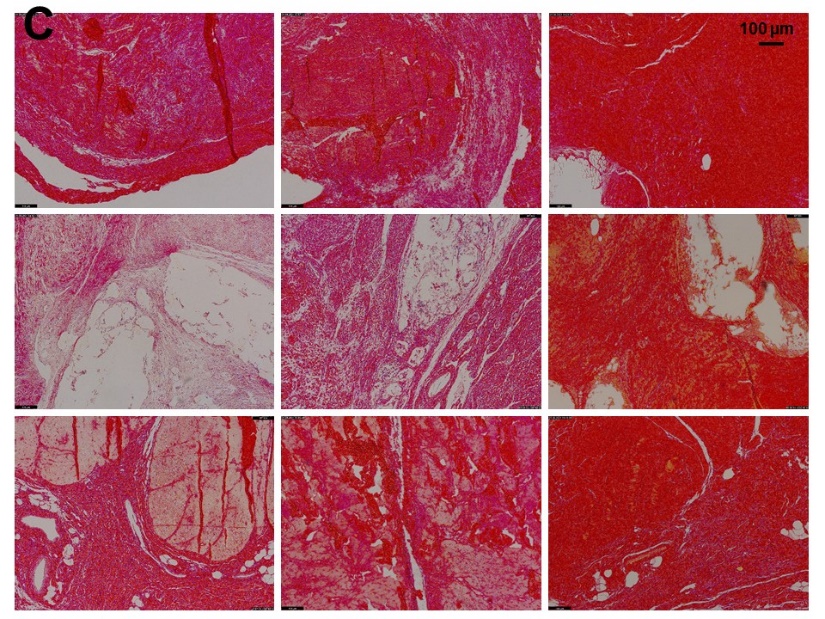


**SI Figure 1**: Adhesion formation as assessed by histology via contact region to the surrounding tissue. Adhesions were determined for 12 weeks. For comparison, adhesion extent for time points 3 and 6 weeks are shown as well. They were previously determined with the same protocol  (Meier Buergisser *et al.*, 2014). Quantitative determination: percentage of contact region (**A**): typical Picosirius Red stained cross-sections (**B**) with rectangles (white dashed) to show areas of higher magnification (**C**). *Key*: DP = DegraPol, NT (not treated). 1-way analysis of variance (ANOVA) was performed for the normally distributed data. Pairwise comparison probability is indicated by stars, with p-values: p < 0.05 (*); p < 0.01 (**) and p < 0.001 (***). Scale bars indicate 500 µm (**B**) and 100 µm (**C**), respectively.

**
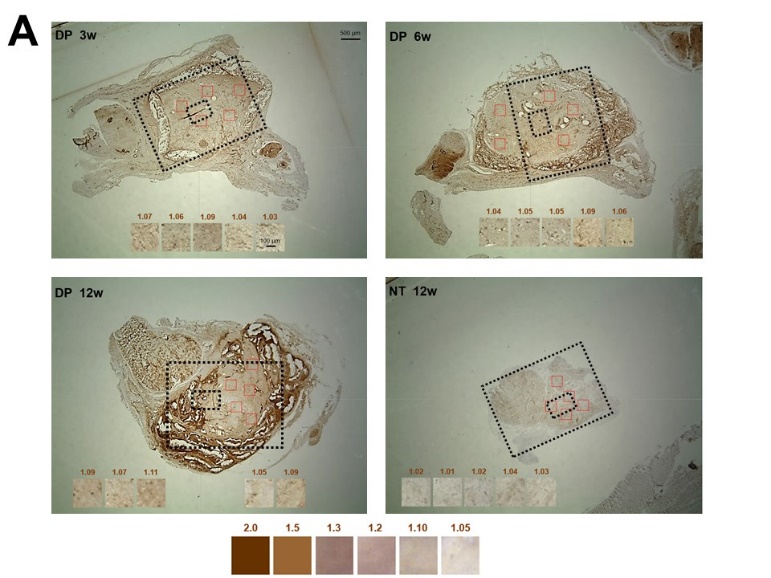
**

**
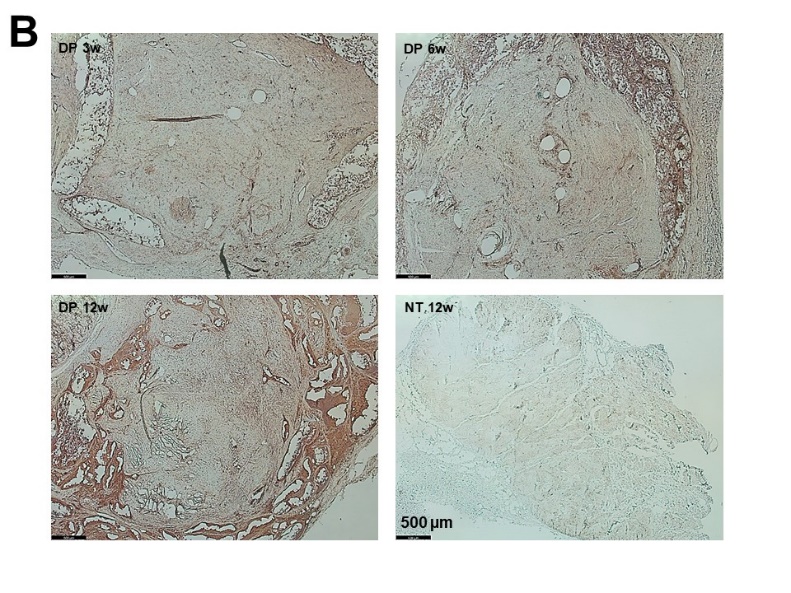
**

**
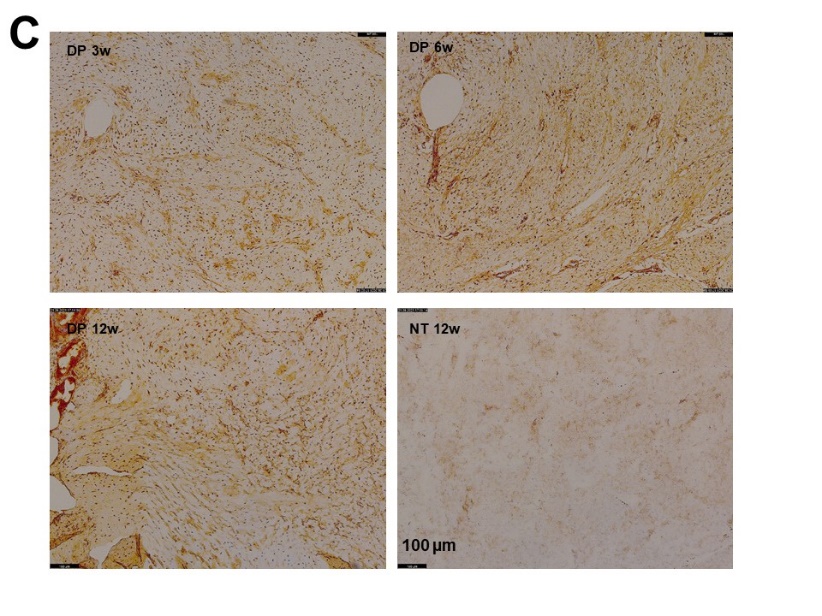
**

**SI Figure 2**: Determination of brown intensity in immunohistochemically PAR-2 stained sections; for each group (3 time points, 2 conditions (DP and NT), 5 FOVs per section were taken, in total 15 FOVs per group. FOVs were denoted by red squares, with magnifications below) (**A**). The red-to-green-ratio of PAR-2-staining is given above each FOV. Below: reference scale for whole brown spectrum with red-to-green ratios. Big black dashed areas in (**A**) show regions magnified in (**B**); small black dashed areas in (**A**) depict regions magnified in (**C**). Key: DP = DegraPol, w= week.


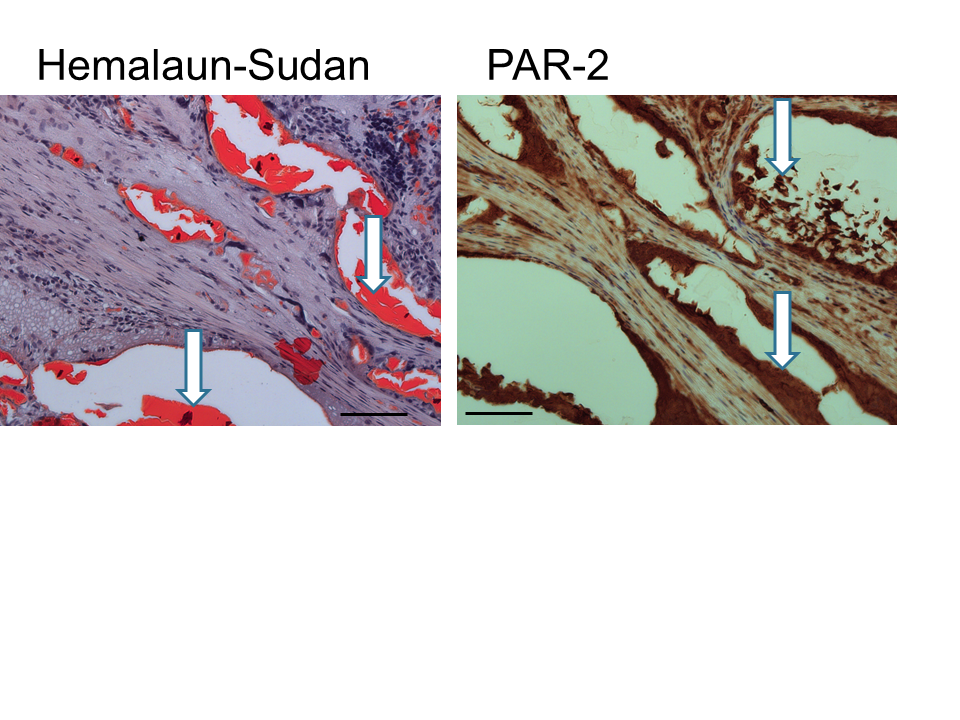


**SI Figure 3**: Comparison of Hemalaun-Sudan stained section and PAR-2 stained section of tube-treated rabbit Achilles tendon at 12 weeks. Arrows show that the degrading polymer DegraPol (DP) is properly intruded by novel tendon tissue and breaks apart. Important, PAR-2 immunohistochemical staining stains the DP dark brown, but not the tendon tissue with the tenocytes, respectively, which can be compared to the red staining of DP in the Hemalaun-Sudan stained section. Scale bars indicate 50 µm.


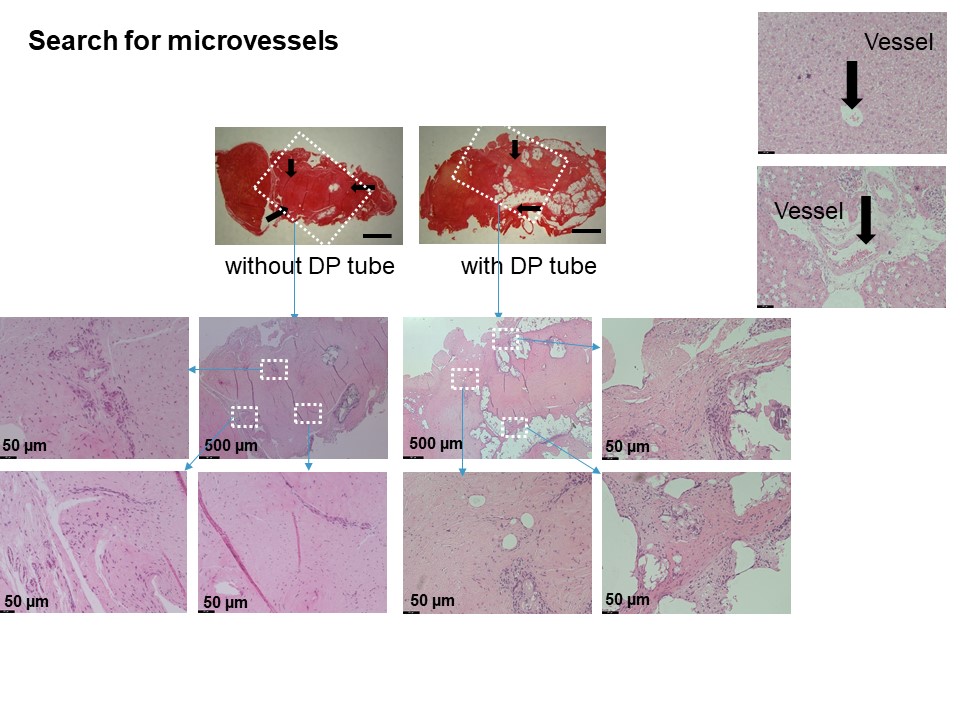


**SI Figure 4**: Search for microvessel formation 12 weeks post-operation in H&E stained sections. Localization of the areas (white dashes lines) is given by Picosirius stained sections. There were no microvessels visible, neither in the operated tendons without tube nor in the ones with tube. Positive controls are depicted in the right upper corner with black arrows showing typical vessels with erythrocytes in rabbit tissue.

**Reference cited in the Supporting Information**

Meier Buergisser G, Calcagni M, Muller A, Bonavoglia E, Fessel G, Snedeker JG, Giovanoli P, Buschmann J (2014) Prevention of peritendinous adhesions using an electrospun DegraPol polymer tube: a histological, ultrasonographic, and biomechanical study in rabbits. BioMed research international: 656240.
